# Pyomyositis Affecting the Paraspinal and Iliacus Muscles in a Patient With Staphylococcus aureus Urinary Tract Infection

**DOI:** 10.7759/cureus.19003

**Published:** 2021-10-24

**Authors:** Hafsa Javed, John Kamara, Samson O Oyibo

**Affiliations:** 1 General Medicine, Peterborough City Hospital, Peterborough, GBR; 2 Diabetes and Endocrinology, Peterborough City Hospital, Peterborough, GBR

**Keywords:** iliacus muscle, paraspinal abscess, fever, back pain, pyomyositis, staphylococcus aureus, urinary tract infection, bacterial pyomyositis

## Abstract

We report a case of a 63-year-old man who presented with a four-day history of fever, night sweats, and left lower back pain, which radiated down to his left buttock and leg. He also had a short-lived episode of chest pain and breathlessness. He had a medical history of chronic back pain, which had been diagnosed as sciatica three years ago. Initial investigations revealed raised inflammatory markers due to a *Staphylococcus aureus* urinary tract infection. Despite treatment, his inflammatory markers did not improve and the left lower back pain persisted. A magnetic resonance imaging scan demonstrated features consistent with pyomyositis of the left lumbar erector spinae (paraspinal) and iliacus muscles. After prolonged antibiotic therapy, his symptoms completely resolved. Pyomyositis is a rare tropical infection of the skeletal muscles most commonly caused by *Staphylococcus aureus*. Risk factors include trauma and immunosuppression. This case highlights a nearly missed diagnosis of paraspinal and iliacus pyomyositis in patients with a background history of chronic lower back pain. Early diagnosis and treatment are pivotal in preventing serious complications such as septicemia and multi-organ failure.

## Introduction

Pyomyositis is a rare, subacute, deep muscle infection due to transient bacteremia, often seen in tropical countries. *Staphylococcus aureus* and *Streptococcus pyogenes* are the most common causative organisms [1]. Fungi, viral, and parasitic infections have also been implicated [2]. The pathogenesis of pyomyositis involves transient bacteremia in the setting of muscle injury. There is usually a history of preceding trauma or strain to the affected muscles. Trauma may also lead to hematoma formation that may be seeded during transient bacteremia. Large lower extremity muscles are commonly affected but pyomyositis can affect any skeletal muscle group [2].

Pyomyositis affecting the iliacus muscle, which is deep in the pelvic fossa, is rare and often misdiagnosed as septic arthritis of the hip. There have been several reports mainly in children [3]. Pyomyositis affecting the paraspinal muscles (cervical, thoracic, lumbar, or psoas) is also rare, making up less than 4% of pyomyositis cases. Paraspinal pyomyositis is also more common in children and young adult men [4]. A delay in diagnosing paraspinal and iliacus pyomyositis can result in osteomyelitis of the adjacent spinal and pelvic bones, retroperitoneal abscess, sepsis, and occasionally death [4].

Pyomyositis can be diagnostically challenging due to the deep location of the affected muscles and non-specific symptoms [1,2]. A diagnosis of pyomyositis affecting the deep muscles of the back and pelvis can be missed in patients who also have a background history of chronic back pain and sciatica. We describe a male patient with a background history of chronic lower back pain, who presented with fever, night sweats, and severe back pain. It transpired that he had a case of pyomyositis affecting his paraspinal and iliacus muscles, accompanying a *Staphylococcus aureus* urinary tract infection.

## Case presentation

Medical history and demographics

A 63-year-old male presented with a four-day history of fever, night sweats, and left-sided lower back pain. On the day of attendance to the emergency department, he admitted that he had experienced a short-lived episode of sharp, central chest pain and breathlessness. He had no history of vomiting, abdominal pain, or dysuria. His past medical history included chronic lower back pain radiating to his left buttock and leg, diagnosed as sciatica three years ago. He had no history of foreign travel. He was not on any regular medication and was a non-smoker.

Examination of the patient revealed a raised temperature of 38.1°C, respiratory rate of 20 breaths per minute, oxygen saturation of 95% on air, a pulse rate of 81 beats per minute, and blood pressure of 113/74 mmHg. Chest, cardiac and abdominal examinations were unremarkable. He had mild left buttock tenderness and pain on weight-bearing.

Investigations

Investigations revealed a normal white cell count but raised C-reactive protein (CRP) and D-dimer levels (Table 1). Renal function, liver function, and troponin T levels were normal. Because of the short-lived history of chest pain and breathlessness, the patient had a computerized tomography pulmonary angiogram, which excluded pulmonary embolism and lung consolidation. An electrocardiogram was also normal. An ultrasound scan of the kidneys, ureter, and bladder was normal. Spot urinalysis was positive for nitrites, red blood cells, and white blood cells, indicating a urinary tract infection. Subsequent urine culture yielded a white blood cells count of 34 per mL, red blood cells of 11 per mL, and more than 10^5^ per mL of the *Staphylococcus aureus* bacteria, which was sensitive to flucloxacillin, nitrofurantoin, trimethoprim, and mupirocin (standard methodology using the latex agglutination and microtube coagulase test [Pro-Lab Diagnostics, United Kingdom]).

**Table 1:** Initial blood tests and results.

Blood test	Result	Reference range
Sodium (mmol/L)	137	132-145
Potassium (mmol/L)	4.1	3.4-5.1
Chloride (mmol/L)	103	97-110
Creatinine (mmol/L)	88	45-84
Urea (mmol/L)	9.7	2.5-7.8
Troponin T (ng/L)	10	<14
Alkaline phosphatase (U/L)	142	30-130
Total bilirubin (mmol/L)	17	<21
Total protein (g/L)	66	60-80
Albumin (g/L)	36	35-50
C-reactive protein (mg/L)	323	<5
Hemoglobin (g/L)	133	115-165
White cell count (10^9^/L)	9.6	4.0-11.0
Platelet count (10^9^/L)	184	150-400
D-dimer (ng/ml)	635	<243

**Figure 1:**
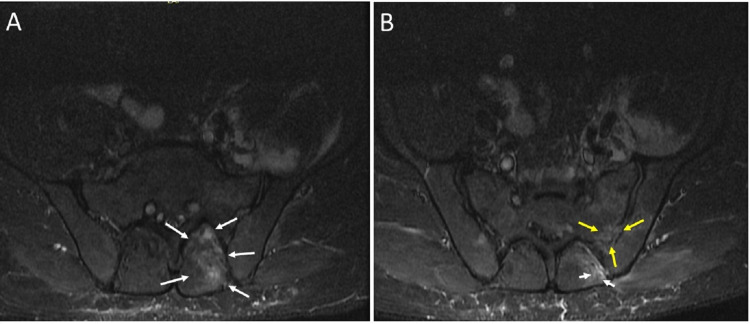
Magnetic resonance imaging (MRI) scan of the lumbosacral spine demonstrating inflammatory changes. (A) Pyomyositis of the left-sided erector spinae muscle (white arrows), and (B) pyomyositis of the left iliacus muscle (yellow arrows) and erector spinae muscle (white arrows).

On day four of admission, he continued to have temperature spikes. His back pain, which hitherto had not been regarded as clinically significant, had become worse. Repeat examination revealed tenderness over the second to fourth lumbar vertebra and inability to perform the straight leg raise test on the left side. A magnetic resonance imaging (MRI) scan of the lumbosacral spine demonstrated inflammatory changes in the left-sided erector spinae muscles extending from the fifth lumbar to the second sacral vertebra and the left iliacus muscle consistent with paraspinal and iliacus pyomyositis (Figure 1). There was no evidence of accompanying discitis.

Further investigations performed to look for other causes of pyomyositis were normal (Table 2). A diagnosis of paraspinal and iliacus pyomyositis possibly associated with a *Staphylococcus aureus* urinary tract infection was made.

**Table 2:** Results of further investigations.

Further investigations	Result
Blood culture	Negative
QuantiFERON-TB Gold test for mycobacterium tuberculosis	Negative
Urine for legionella and streptococcal antigen	Negative
Virology for Epstein-Barr, cytomegalovirus, hepatitis, and human immunodeficiency	Negative
Anti-nuclear antibody tests	Negative
Creatinine phosphokinase	Normal
Biopsy of the affected erector spinae muscles (performed on day 14 of antibiotic treatment)	Negative

Treatment

The patient was initially treated with intravenous co-amoxiclav for a urinary tract infection. However, on day two of admission, his temperature remained elevated at 37.8°C and CRP rose to 426 mg/L. The antibiotic regimen was, therefore, escalated to intravenous piperacillin/tazobactam (Tazocin) after obtaining advice from our microbiologist.

Outcome and follow-up

The patient received three weeks of intravenous antibiotics. His repeat urine culture was sterile. His CRP gradually fell to 29 mg/L by day 17 of admission. His left-sided back, buttock, and leg pain resolved completely. He went home on day 18 with a three-week course of oral antibiotics. He remained well thereafter.

## Discussion

Pyomyositis affects all age groups, although young males are most susceptible [5]. In the temperate region, pyomyositis is prevalent in immunocompromised individuals. In patients who are not immunocompromised, there may be difficulty or delay in detecting the disease without high suspicion. Other predisposing factors include intravenous drug abuse and concurrent infection [5].

Fever, myalgia, and localized tenderness are the classical symptoms of this condition. Three stages of pyomyositis have been described [6]. The initial invasive stage is characterized by subacute onset of fever and pain but minimal systemic symptoms. The suppurative (purulent) stage usually develops between the second and third week with abscess formation in the muscle; most cases present at this stage. The classical local signs of an abscess may be absent because the overlying muscle is tense. Untreated cases may progress to the late septicemic stage where dissemination occurs, resulting in bacteremia, metastatic abscess formation, septic shock, and acute multi-organ failure. The septicemic stage can also occur early or late in the disease [6].

Laboratory investigations demonstrate leukocytosis, anemia, and raised inflammatory markers. An MRI scan detects muscle inﬂammation, even in the early stages of pyomyositis before frank abscess formation. Aspiration of pus from the muscle or a muscle biopsy for microbiological examination is the gold standard for diagnosis, though maybe sterile in 15%-30% of cases. Muscle biopsy also aids in excluding other conditions, such as osteosarcoma and toxoplasmosis [6]. Treatment of pyomyositis involves a long course of antibiotics based on local protocols and sensitivity with surgical drainage of any abscess [7]. Infectious disease specialist/microbiologist's advice should be sorted, as was done in our case. Penicillins are the first line of therapy for most patients. In those with penicillin allergy, first-generation cephalosporins are recommended, while vancomycin is preferred for methicillin-resistant *Staphylococcus aureus* [7].

Pyomyositis affecting the paraspinal and pelvis floor muscle groups is rare and can be difficult to diagnose because of the deep location [3,4,8]. In the lumbosacral region, the posterior paraspinal muscles include the erector spinae and multifidus muscles, while the iliacus muscle lies in the iliac fossa of the pelvic bone. The fact that pyomyositis is due to transient bacteremia rather than a local extension of infection may explain why pyomyositis occurs far from the original source of infection and can affect more than one muscle group simultaneously. Our patient had acute pyomyositis affecting two separate muscle groups (paraspinal and iliacus) accompanying a *Staphylococcal aureus* urinary tract infection. The chronic trauma or strain to the affected muscle groups, as indicated by the history of chronic back pain, may have been the underlying risk factor. Extra strain or trauma to both muscle groups may have rendered them susceptible to being seeded during transient bacteremia. Ironically, the same background history of chronic back pain resulted in us not attaching much clinical importance to the severe back pain at initial presentation, as it was assumed that this was an exacerbation of his usual chronic lower back pain. The diagnosis of paraspinal and iliacus pyomyositis could have been missed in this case, had the back pain not been recognized as clinically important.

There have been reported cases of *Staphylococcal* bacteria-related paraspinal pyomyositis occurring in the setting of a urinary tract infection: these have been in patients who also have diabetes mellitus [9,10]. The blood culture sample from our patient did not yield any bacteria. However, the blood sample was taken 48 hours after the commencement of antibiotic therapy. The patient could have had transient bacteremia earlier on in the disease process. The paraspinal muscle biopsy specimen did not demonstrate any organisms. Of note, the muscle biopsy was performed after 14 days of intravenous antibiotic therapy. Although we have no microbiological confirmation that the paraspinal and iliacus pyomyositis was related to the *Staphylococcal aureus *urinary tract infection, the coexistence of both conditions seems more than a mere coincidence.

## Conclusions

We have presented a case of pyomyositis affecting the paraspinal and iliacus muscle groups in a man who had a *Staphylococcus aureus* urinary tract infection. Pyomyositis affecting the deep back and pelvic muscles can be diagnostically challenging due to the deep location of the affected muscles and the non-specific symptoms. The diagnosis can be delayed or missed in the presence of a background history of chronic lower back pain. A diagnosis of paraspinal pyomyositis should be considered in patients who present with a combination of muscular back pain (whether acute or chronic), raised inflammatory markers, and fever.
